# Multi-Session Transcranial Direct Current Stimulation (tDCS) Elicits Inflammatory and Regenerative Processes in the Rat Brain

**DOI:** 10.1371/journal.pone.0043776

**Published:** 2012-08-22

**Authors:** Maria Adele Rueger, Meike Hedwig Keuters, Maureen Walberer, Ramona Braun, Rebecca Klein, Roland Sparing, Gereon Rudolf Fink, Rudolf Graf, Michael Schroeter

**Affiliations:** 1 Department of Neurology, University Hospital of Cologne, Cologne, Germany; 2 Center for Experimental Medicine, University Hospital of Cologne, Cologne, Germany; 3 Max Planck Institute for Neurological Research, Cologne, Germany; 4 Cognitive Neurology Section, Institute of Neuroscience and Medicine (INM-3), Research Centre Juelich, Juelich, Germany; Universitätsklinikum Carl Gustav Carus an der Technischen Universität Dresden, Germany

## Abstract

Transcranial direct current stimulation (tDCS) is increasingly being used in human studies as an adjuvant tool to promote recovery of function after stroke. However, its neurobiological effects are still largely unknown. Electric fields are known to influence the migration of various cell types *in vitro*, but effects *in vivo* remain to be shown. Hypothesizing that tDCS might elicit the recruitment of cells to the cortex, we here studied the effects of tDCS in the rat brain *in vivo*. Adult Wistar rats (n = 16) were randomized to either anodal or cathodal stimulation for either 5 or 10 consecutive days (500 µA, 15 min). Bromodeoxyuridine (BrdU) was given systemically to label dividing cells throughout the experiment. Immunohistochemical analyses *ex vivo* included stainings for activated microglia and endogenous neural stem cells (NSC). Multi-session tDCS with the chosen parameters did not cause a cortical lesion. An innate immune response with early upregulation of Iba1-positive activated microglia occurred after both cathodal and anodal tDCS. The involvement of adaptive immunity as assessed by ICAM1-immunoreactivity was less pronounced. Most interestingly, only cathodal tDCS increased the number of endogenous NSC in the stimulated cortex. After 10 days of cathodal stimulation, proliferating NSC increased by ∼60%, with a significant effect of both polarity and number of tDCS sessions on the recruitment of NSC. We demonstrate a pro-inflammatory effect of both cathodal and anodal tDCS, and a polarity-specific migratory effect on endogenous NSC *in vivo*. Our data suggest that tDCS in human stroke patients might also elicit NSC activation and modulate neuroinflammation.

## Introduction

Transcranial direct current stimulation (tDCS) has long been known to induce long-lasting alterations of cortical excitability both in experimental animals [1] and humans [2]. It has been used as a reversible neuromodulation technique to study the behavioral impact of distinct neural networks in experimental animals [3]. Given further results demonstrating that non-invasive brain stimulation enhances the effects of motor learning in healthy volunteers [4], [5], recent studies have tried to exploit the therapeutic potential of the method for various neurological and psychiatric disorders. Studies in human stroke patients have shown tDCS to be a promising therapeutic intervention to ameliorate motor deficits [6], aphasia [7], and neglect [8], suggesting that tDCS may become a new adjuvant tool to promote recovery of function after stroke [9]. Although tDCS is already widely used in human studies, the basic mechanisms of its action remain incompletely explored, and details about the neurobiological effects of tDCS are still unknown (for comprehensive reviews, see [10], [11]. The long-lasting effects on cortical excitability outlasting the actual stimulation (‘after-effects’) seem to depend on alterations of resting membrane potentials and NMDA-receptor dependent synaptic plasticity [12]. However, it was recently reported that the impact of tDCS exceeds those primary electrophysiological effects, as tDCS was shown to modulate cerebral blood flow in a polarity-specific way, potentially via neuro-vascular coupling [13]. Moreover, recent theoretical calculations suggested alterations in the transmembrane potential of glial cells rather than neurons, challenging the view that the effects of tDCS effects are neuronally driven, and refocusing the search for its neurobiological effects on non-neuronal cell populations [14]. Studies *in vitro* have shown that electric fields induce cultured cells to migrate, a phenomenon referred to as galvanotaxis [15]. This has been demonstrated for various types of cells, among them fibroblasts [16], granulocytes [17], and keratinocytes [18]. Interestingly, recent reports have also found rodent neural progenitor cells [19], [20], human embryonic stem (ES) cells [21], and human ES-cell derived neural stem cells [22] to migrate in the electric field *in vitro*. However, whether such effects can also be observed *in vivo* has not yet been investigated. We hypothesized that tDCS may attract cells inflicted in reparative responses after stroke. This is a proof of principle study, and in order not to miss subtle effects, we stimulated adult rats with high current density, but, importantly, below lesion threshold.

**Figure 1:**
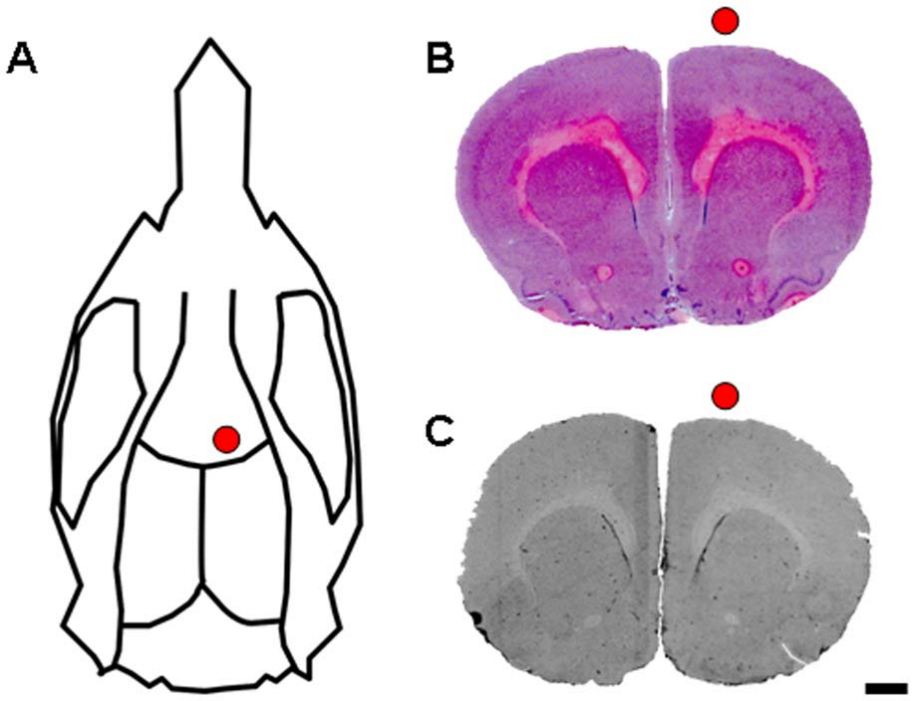
Multi-session transcranial DCS with defined parameters does not cause a cerebral lesion or astrogliotic scar. (A) For multi-session tDCS, an epicranial electrode was mounted as schematically depicted (red circle). (B) H&E staining confirmed an intact cortex after multi-session focal tDCS, and (C) no astrogliotic scarring was detected by GFAP staining (scale bar  = 1 mm).

## Materials and Methods

### Animals and Surgery

All animal procedures were in accordance with the German Laws for Animal Protection and were approved by the local animal care committee and local governmental authorities. Spontaneously breathing male Wistar rats weighing 290–330 g were anesthetized with 5% isoflurane and maintained with 2.5% isoflurane in 65% / 35% nitrous oxide / oxygen. Throughout surgical procedures, body temperature was maintained at 37.0°C with a thermostatically controlled heating pad.

**Figure 2:**
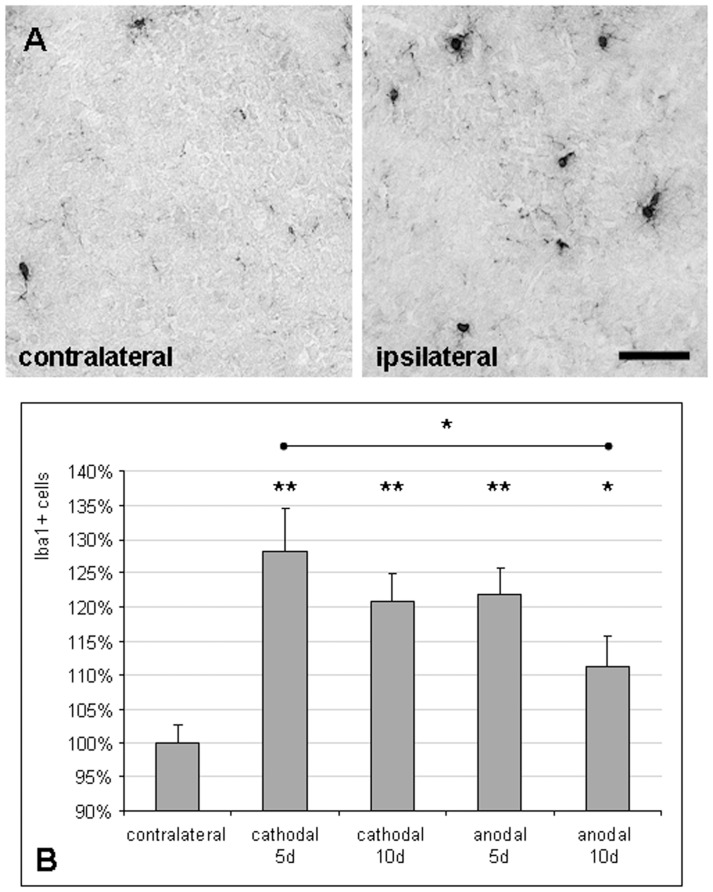
Multi-session tDCS activates cortical microglia. (A) The density of activated microglia identified by Iba1 immunoreactivity was increased in the cortex ipsilateral to multi-session tDCS (scale bar  = 0.05 mm). (B) This increase in Iba1+ cells compared to the respective contralateral hemisphere was most pronounced early after cathodal tDCS, but also present after anodal stimulation; effects decreased with time (means ± SEM; *p = 0.05, **p<0.01).

#### Multi-session transcranial direct current stimulation

An epicranial electrode with a defined contact area of 3.5 mm^2^ was mounted onto the intact skull using non-toxic glass ionomer luting cement (Ketac Cem Plus, 3 M ESPE, Seefeld, Germany) at the following stereotaxic coordinates: bregma AP +2.0 mm, ML +2.0 mm. After electrode placement, the skin around the electrode was closed with sutures, and the electrode left in place for the entire experiment. The counter electrode was placed on the rat's ventral thorax. tDCS was applied continuously for 15 min at 500 µA using a constant current stimulator (CX-6650, Schneider Electronics, Gleichen, Germany), according to the protocol by Liebetanz et al [23]. The chosen parameters led to a charge density (current x time / area) of 128571 C / m^2^. tDCS was performed under anesthesia to avoid dislocation of the cable. At the first day of the experiment, animals were randomized to either anodal (n = 6) or cathodal (n = 10) tDCS. tDCS was repeated daily using the same parameters for a total of 5 consecutive days, followed by a tDCS-free interval of 3 days. Eight out of 16 animals were sacrificed for histology at that time point, while another 8 animals were subjected to tDCS for 5 more days (n = 3 anodal, n = 5 cathodal), resulting in a total of 10 days of tDCS. After each procedure, all animals were allowed to recover from anesthesia and were put back into their home cages, where they were given access to food and water *ad libitum*.

**Figure 3:**
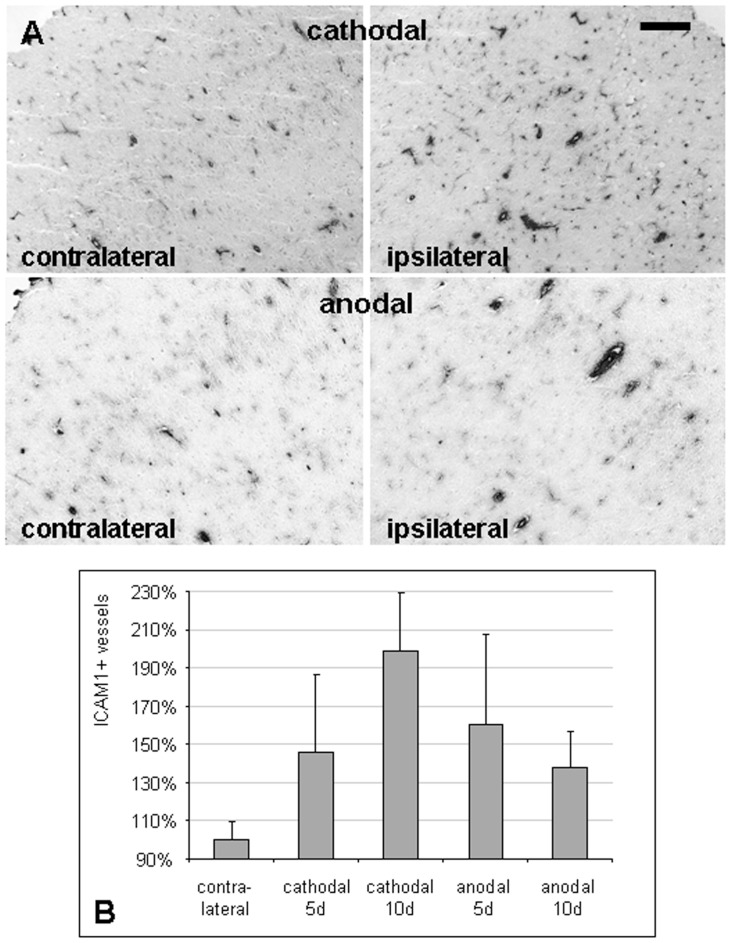
Multi-session tDCS leads to ICAM1 upregulation. (A) ICAM1 immunoreactivity was increased in the ipsilateral hemisphere after multi-session cathodal or anodal tDCS, (scale bar  = 0.2 mm). (B) ICAM1+ vessels trended to increase with time upon cathodal tDCS, while anodal tDCS caused only a transient increase (note that differences were not statistically significant; means ± SEM).

#### BrdU injections

In all animals, the tracer bromodeoxyuridine (BrdU) was injected intraperitoneally for the duration of the experiment, starting on the first day of tDCS, at a concentration of 50 mg/kg per injection, as described previously [24]. Animals receiving 5 sessions of tDCS were injected with 50 mg/kg BrdU per injection daily just prior to each tDCS session. Animals receiving 10 sessions of tDCS were injected every other day. This regime resulted in a cumulative dose of 250 mg/kg BrdU per animal.

**Figure 4:**
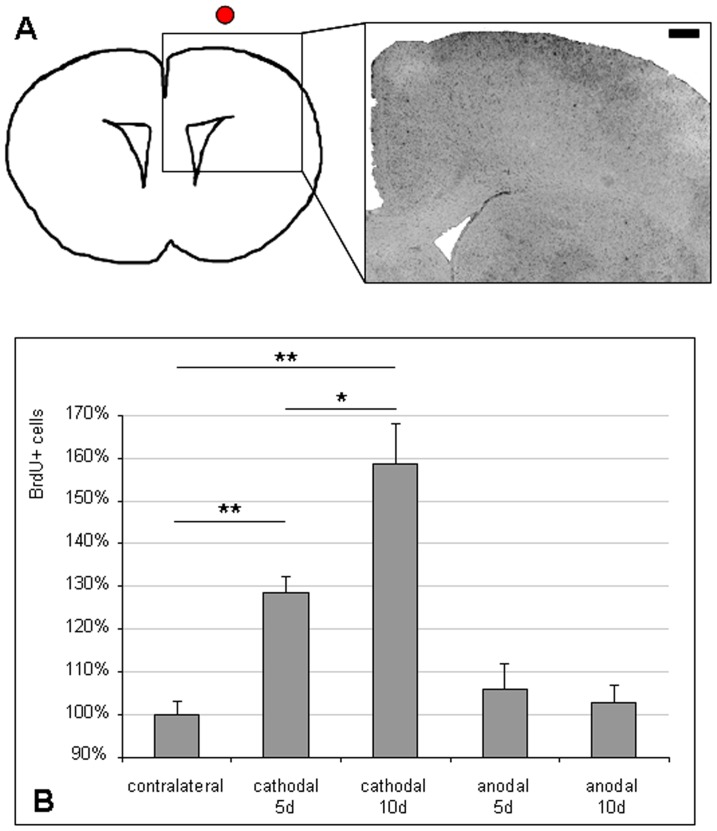
Cathodal tDCS increases the number of proliferating cells in the cortex. (A) After multi-session cathodal tDCS, the number of proliferating cells identified by BrdU-incorporation was increased in the ipsilateral hemisphere (scale bar  = 0.5 mm). (B) This effect was only observed with cathodal stimulation. Anodal tDCS had no effect on proliferation in the cortex (means ± SEM; **p<0.01, *p<0.05).

### Immunohistochemistry

Three days after the last tDCS session, rats were deeply anesthetized and decapitated. The brains were rapidly removed, frozen in isopentane at −40°C, and stored at −80°C prior to further histological and immunohistochemical processing. Ten µm thick adjacent serial coronal brain sections were cut at 500 µm intervals. H&E staining was performed according to standard protocols to detect any cortical lesion due to tDCS. For immunohistochemistry, sections were fixed with either 4% paraformaldehyde (for Iba1, BrdU, and Hes3-stainings), or with 100% acetone (for ICAM1-stainings). For antigen-retrieval prior to BrdU-staining, sections were microwave-heated in 0.01 M citrate buffer, pH 6.0, for 5 min, followed by 2N HCl at 37°C for 30 min. The following antibodies were used: anti-Iba1 (rabbit polyclonal, cat# 019-19741, dilution 1∶1000, Wako, Neuss, Germany); anti-ICAM-1 (mouse monoclonal, anti-rat CD54, cat-# MCA773GA, dilution 1∶500, AbD Serotec, Puchheim, Germany); anti-BrdU (mouse monoclonal, cat-# B2531, dilution 1∶100, Sigma, Munich, Germany); anti-Hes3 (rabbit polyclonal, cat-# sc-25393, dilution 1∶1000, Santa Cruz Biotechnology, Heidelberg, Germany). For visualization of all antibodies, the ABC Elite kit (Vector Laboratories, Burlingame, CA, USA) with diaminobenzidine (Sigma, Munich, Germany) as the final reaction product was used.

**Figure 5:**
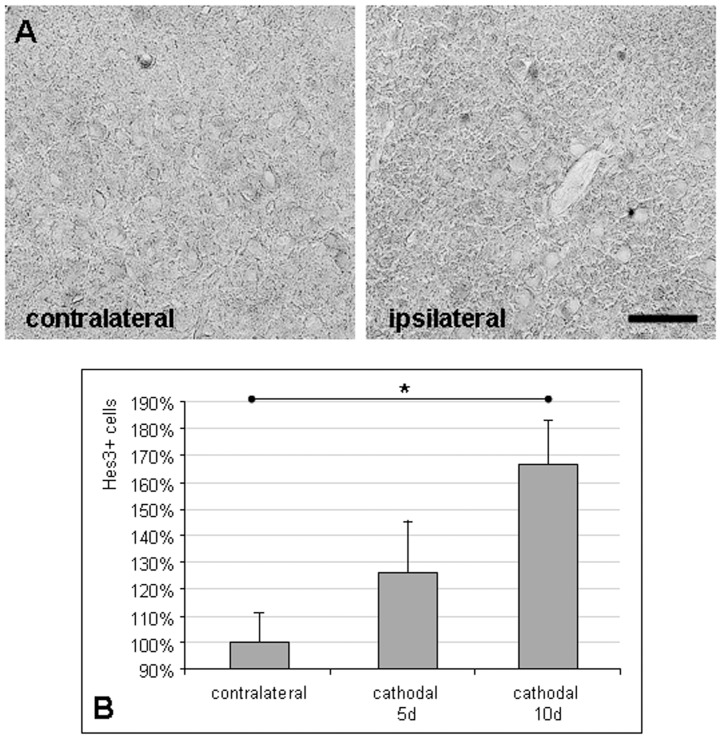
Cathodal tDCS increases the number of Hes3-positive neural stem cells in the cortex. (A) The density of NSC identified by Hes3 immunoreactivity was increased in the cortex ipsilateral to multi-session tDCS (scale bar  = 0.05 mm). (B) The number of Hes3-positive NSC increased in the ipsilateral hemisphere with the duration of cathodal tDCS (means ± SEM; *p<0.05).

### Quantification and statistical analysis

To determine the number of Iba1-positive, BrdU-positive and Hes3-positive cells and of ICAM1-positive vessels in the cortex, 20 coronal sections at 500 µm intervals were stained with the respective antibody. Using a Zeiss Axiophot microscope with a 40x objective, 7 images of adjacent fields of view (FOV) of the cortex were taken of each hemisphere, and of each section. The number of positive cells in the ipsilateral, stimulated hemisphere was divided by the corresponding number in the contralateral, control hemisphere. Mean values and standard errors of the mean of these ratios were calculated for each group of animals that had received the same treatment.

Descriptive statistics and Student's t-tests were performed with Microsoft Excel 2003 (Microsoft Corp.); statistical significance was set at the less than 5% level (p<0.05).

## Results

### Multi-session tDCS with defined parameters did not cause a cerebral lesion or astrogliotic scar

Rats were subjected to 15 min sessions of tDCS using a 3.5 mm^2^ epicranial electrode and a current of 500 µA (**Fig. 1A**). This protocol resulted in a charge density (current x time / area) of 128571 C / m^2^ per single tDCS session. No epileptic seizures or neurological deficits were induced by tDCS.

After 5 or 10 sessions of anodal or cathodal tDCS, histological analyses revealed that none of the rats had suffered a cortical lesion (**Fig. 1B**). As assessed by GFAP immunoreactivity, tDCS had neither caused an astrogliotic scar in the stimulated cortex (**Fig. 1C**).

#### Multi-session tDCS activated cortical microglia

To assess the effects of tDCS on innate neuroinflammatory processes, rat brains were stained for Iba1, labelling activated microglia. In the ipsilateral (stimulated) cortex, the number of Iba1-positive cells was increased after both cathodal and anodal stimulation (**Fig. 2A**). This upregulation of Iba1-positive microglia by 28% was most pronounced early (5 days) after cathodal tDCS (p<0.01) and decreased again with time; anodal tDCS also caused early microglial activation, but to a lesser extent (**Fig. 2B**).

To explore a secondary involvement of adaptive immunity, we investigated the endothelial intercellular adhesion molecule-1 (ICAM1), involved in leukocyte transmigration through the blood-brain barrier. After 5 days, ICAM-1 was increased by trend in the ipsilateral (stimulated) cortex, irrespective of the polarity (**Fig. 3A**). While cathodal stimulation tended to further increase ICAM1-positive vessels with time, the trend was only transient after anodal tDCS (**Fig. 3B**).

#### Cathodal tDCS increased the number of NSC in the cortex

During the 5 or 10 days of multi-session tDCS, animals were repeatedly injected i.p. with BrdU to label proliferating cells. The protocol ensured that all animals received the same cumulative dose of BrdU, regardless of the duration of the experiment. BrdU-positive cells were counted in the ipsilateral cortex and compared to the contralateral side (**Fig. 4A**). Cathodal stimulation of 5 days significantly increased the number of BrdU-positive cells by 29% (p<0.01). Cathodal tDCS for 10 days increased BrdU-positive cells by 59% (p<0.01), indicating a significant effect of the number of tDCS sessions (p<0.05). In contrast, anodal tDCS for 5 or 10 days did not have an effect on the number of BrdU-positive cells in the cortex (**Fig. 4B**).

To further investigate what subpopulations of potentially proliferating cells were expanded by cathodal tDCS, brains were stained for Hes3 as a marker of endogenous NSC (**Fig. 5A**). The number of Hes3-positive cells was increased by trend in the ipsilateral (stimulated) hemisphere following 5 days of cathodal tDCS. Increasing the number of tDCS sessions to 10 significantly elevated Hes3-positive NSC numbers by 67% (p<0.05; **Fig. 5B**).

## Discussion

In this study, we investigated the effects of multi-session tDCS on the adult rat brain *in vivo*, focusing on the cellular responses to stimulation. We demonstrate a pro-inflammatory effect of both cathodal and anodal tDCS that tends to be transient. In addition, only cathodal tDCS induced the recruitment of proliferating NSC to the stimulated hemisphere. Our data suggest a polarity-specific migratory effect on endogenous neural stem cells *in vivo*. tDCS is capable of attracting cells inflicted in reparative and regenerative responses to the site of ischemic stroke. Beneficial effects of tDCS may at least result partly from NSC activation and the modulation of neuroinflammation.

In our experimental paradigm, we took great care to ensure that tDCS would not cause any lesion to the brain tissue. In a recent pioneer study, Liebetanz et al. described the relationship between charge density and the occurrence and size of cortical lesions [25]. While we confirmed that the charge density of 128571 C/m^2^ per single tDCS session was not associated with cortical lesions, it should be noted that the charge density used in our study is several orders of magnitude higher than the charge density usually applied in humans (up to 480 C/m^2^). We chose this high charge density – just below the lesion threshold – in order to detect any putatively faint effect of tDCS on cellular processes. Using this high charge density in multi-session tDCS sessions allowed us to detect the expected effects at a magnitude that reached statistical significance. We hypothesize that lower charge densities as applied in humans may have the same effects, albeit to a potentially lesser extent. Focusing on a proof of principle design, we did not titrate the tDCS stimulation density in this study.

We found the numbers of Iba1-positive cells to be increased early after cathodal tDCS, decreasing again with time. Similar findings were obtained after anodal tDCS, albeit to a lesser extent. This rapid and transient activation of resident microglia is typical for an innate neuroinflammatory response to tDCS. ICAM1 expression is a prerequisite of recruitment of haematogenous cells in an antigen specific manner, categorized as adaptive immunity. ICAM-1 was used as an indicator of upcoming adaptive immune responses. It showed a typical later time-course with a maximum at 10 days following cathodal stimulation, while anodal stimulation caused a more transient, unspecific effect. However, those neuroinflammatory processes observed after both cathodal and anodal stimulation could – at least in part – also be caused by thermal effects that were sub-threshold to induce a lesion to the brain tissue.

Based upon work in focal cerebral ischemia, neuroinflammation has already been characterized as a ‘double-edged sword’ with both beneficial and detrimental effects on the prevention of secondary tissue damage, regeneration and recovery [26]. Destructive effects of neuroinflammation include the damage caused by reactive oxygen species and excessive production of pro-inflammatory cytokines by immune cells, beneficial aspects are the containment of necrotic tissue and the induction of a strong regenerative response including the recruitment of endogenous NSC [26]–[28]. Quality, extent and timing of neuroinflammatory processes determine whether manipulating that particular response will be deleterious or therapeutically beneficial. The activation of resident microglia can under some circumstances be neurotoxic [29], especially when the mode of activation triggers a defense-oriented reaction, such as the application of bacterial endotoxin lipopolysaccharide (LPS). But the engagement of microglia can also be neuroprotective, since ablation of microglial cells in stroke mice leads to a significant increase in infarct size [30]. The difference in the role and function of microglia seems to depend on the activating conditions [31]. Interestingly, differentially activated microglia also have opposing effects on NSC: microglia activated by LPS attenuate neurogenesis, while their activation by cytokines associated with T-helper cells promotes neurogenesis [32]. In our model of non-lesional tDCS, we propose that cathodal tDCS induces a local immune response, predominantly involving innate immunity, potentially conferring neuroprotection and attracting endogenous NSC. However, further experiments are needed to characterize the complex neuroinflammatory pattern following tDCS and the putative interplay between NSC and inflammation.

The transcription factor Hes3 was recently identified as a biomarker for a widespread population of quiescent endogenous NSC that is dynamically upregulated upon their activation [33]. Several recent reports have demonstrated the direct effect of electric fields on the migration of stem cells in culture. This has been shown in adult mouse NSC from the subventricular zone [19], adult rat NSC from the hippocampus [34], embryonic rat NSC from the lateral ganglionic eminence [20], human embryonic stem (hES) cells and human induced pluripotent stem (hiPS) cells [21], and hES cell-derived NSC [22]. All of those cell types were reported to migrate towards the cathode of the electric field, except the hiPS cells that moved towards the anode [21]. Accordingly, we here show an upregulation of proliferating NSC in the non-invasively stimulated brain after cathodal tDCS can also be explained by the migration of endogenous NSC towards the cathode. The extent of eNSC accumulation after cathodal tDCS was comparable to that induced pharmacalogically, e.g. via activation of the Notch receptor [33]. Enhancing the (physiological) mobilization of eNSC after injury such as stroke was previously shown to remarkably improve neurological function and recovery [35]–[37]. However, the differentiation of mobilized eNSC into mature neurons that functionally integrate into the damaged circuitry has rarely been observed, and the vast majority of newly generated migrating neuroblasts in ischemic stroke models die by the time they have reached the peri-infarct area [38]. Recent studies have elucidated several mechanisms other than neurogenesis that convey the stem cells' beneficial effects. An important function of eNSC seems to be neuroprotection [33], [39], with neuroprotective effects mediated through several neuroprotective cytokines such as GDNF, VEGF, and Shh [35], [40], [41]. Other functions of eNSC in regeneration involve the suppression of inflammation in the damaged tissue, and clearance of debris in the injured area such as the peri-infarct tissue [42], [43].

Although BrdU-positive proliferating cells could at least in part constitute microglia, the extent of their upregulation and their exclusive appearance after cathodal tDCS makes it likely that at least the majority of those proliferating cells are not microglia. Furthermore, we found the number of Hes3-positive cells to increase with the number of cathodal tDCS session, further confirming an effect on NSC. In addition to the direct effect of the electric field on NSC migration, a second mechanism for the activation of cortical NSC is conceivable: tDCS was recently shown to modulate cerebral blood flow in a polarity-specific way, with an increase after anodal and decrease after cathodal stimulation, lasting for at least 30 min after tDCS [13]. Transient ischemic stress caused by a brief decrease in blood flow is well known to induce the proliferation of endogenous NSC, conferring a certain ischemic tolerance in the process [44].

This study provides evidence that electric stimulation modulates responses of non-neuronal cells in the brain, and relevantly contributes to our scarce knowledge about the neurobiological effects of tDCS. tDCS both attracts endogenous NSC and activates innate immune responses. From the clinical point of view, applying tDCS after stroke may help to locally augment endogenous NSC known to promote neuroprotection and repair.
